# Average Is Optimal: An Inverted-U Relationship between Trial-to-Trial Brain Activity and Behavioral Performance

**DOI:** 10.1371/journal.pcbi.1003348

**Published:** 2013-11-07

**Authors:** Biyu J. He, John M. Zempel

**Affiliations:** 1 National Institute of Neurological Disorders and Stroke, National Institutes of Health, Bethesda, Maryland, United States of America; 2 Departments of Neurology and Pediatrics, Washington University School of Medicine, St. Louis, Missouri, United States of America; Indiana University, United States of America

## Abstract

It is well known that even under identical task conditions, there is a tremendous amount of trial-to-trial variability in both brain activity and behavioral output. Thus far the vast majority of event-related potential (ERP) studies investigating the relationship between trial-to-trial fluctuations in brain activity and behavioral performance have only tested a monotonic relationship between them. However, it was recently found that across-trial variability can correlate with behavioral performance independent of trial-averaged activity. This finding predicts a U- or inverted-U- shaped relationship between trial-to-trial brain activity and behavioral output, depending on whether larger brain variability is associated with better or worse behavior, respectively. Using a visual stimulus detection task, we provide evidence from human electrocorticography (ECoG) for an inverted-U brain-behavior relationship: When the raw fluctuation in broadband ECoG activity is closer to the across-trial mean, hit rate is higher and reaction times faster. Importantly, we show that this relationship is present not only in the post-stimulus task-evoked brain activity, but also in the pre-stimulus spontaneous brain activity, suggesting anticipatory brain dynamics. Our findings are consistent with the presence of stochastic noise in the brain. They further support attractor network theories, which postulate that the brain settles into a more confined state space under task performance, and proximity to the targeted trajectory is associated with better performance.

## Author Summary

The human brain is notoriously “noisy”. Even with identical physical sensory inputs and task demands, brain responses and behavioral output vary tremendously from trial to trial. Such brain and behavioral variability and the relationship between them have been the focus of intense neuroscience research for decades. Traditionally, it is thought that the relationship between trial-to-trial brain activity and behavioral performance is monotonic: the highest or lowest brain activity levels are associated with the best behavioral performance. Using invasive recordings in neurosurgical patients, we demonstrate an inverted-U relationship between brain and behavioral variability. Under such a relationship, moderate brain activity is associated with the best performance, while both very low and very high brain activity levels are predictive of compromised performance. These results have significant implications for our understanding of brain functioning. They further support recent theoretical frameworks that view the brain as an active nonlinear dynamical system instead of a passive signal-processing device.

## Introduction

An inverted-U relationship is well established between brain function and many neuromodulatory influences, including arousal [1], dopaminergic [2], [3], cholinergic [4] and noradrenergic [5], [6] systems, with both insufficient and excessive levels of neuromodulation causing impaired brain function and performance. Interestingly, the possibility that a similar inverted-U function might exist between trial-to-trial fluctuations of brain activity and behavioral performance is seldom tested (but see [7]–[10]). The vast majority of studies on the relationship between trial-to-trial brain activity and behavior have only investigated a monotonic relationship between them by, for example, comparing trial-averaged brain activity between different categories of behavioral performance (e.g., hits vs. misses) or computing the linear correlation between trial-to-trial brain activity and performance metrics [e.g., reaction times (RTs)]. These methods have been successfully applied to reveal influence on cognition/behavior by both pre-stimulus ongoing brain activity and post-stimulus brain responses in functional magnetic resonance imaging (fMRI) [11]–[15] and magnetoencephalography (MEG) [16], [17] signals from humans, as well as local field potentials (LFP) [18], [19] and neuronal spiking activity from primates [20]–[22].

Recently, it has been increasingly appreciated that across-trial brain variability can be modulated independently from trial-averaged brain activity [23]–[30]. Moreover, behaviorally relevant information can be encoded within across-trial variability but not trial-averaged activity [28], [30]. This phenomenon predicts the existence of a U- or inverted-U relationship between trial-to-trial brain activity and behavioral performance. For example, if trials with fast and slow reaction times are associated with a similar level of trial-averaged activity but fast trials have smaller across-trial variability, as observed previously [28], [30], then brain activity closer to the across-trial mean should be more likely to be associated with fast RTs. Notably, several previous studies have shown that pre-stimulus amplitude of brain oscillations in sensory cortices has an inverted-U relationship with behavioral performance: Intermediate amplitudes predict higher hit rate and faster reaction times [7]–[9]. In light of the prevalent phase-amplitude coupling in the human brain whereby lower-frequency phase modulates higher-frequency power [31], [32], we conjectured that such an inverted-U relationship might also manifest in the raw fluctuations of field potentials, which is dominated by low-frequency activity [31].

Theoretically, the existence of both monotonic and inverted-U relationships between trial-to-trial brain activity and behavioral performance accord with the consideration that there are two sources of brain variability: deterministic and stochastic. The deterministic source of variability arises from spontaneous brain activity related to overall brain functioning that varies from trial to trial [33]. Theoretical work further suggests that small differences in the initial state of a system can be deterministically amplified during responses to task [34]. The second source of variability is stochastic noise: Ion channel behavior is fundamentally indeterminate; ion channel noise contributes to synaptic noise, thence to membrane potential fluctuations and spike generation and propagation [35]–[37]. As inescapable as stochastic noise is, randomness at the behavioral level confers an evolutionary advantage in a competitive ecological environment [35]. As remarked by Alan Turing, “If a machine is expected to be infallible, it cannot also be intelligent” (1947, Lecture to London Mathematical Society).

Germane to the current thesis, the presence of stochastic noise predicts an inverted-U relationship between trial-to-trial brain activity and behavioral performance: Under a large amount of noise, brain activity will be scattered across a wide range; under low noise, activity will remain close to the center “targeted” value. Even though the presence of stochastic noise is evolutionarily advantageous and beneficial in contexts such as stochastic resonance [38], gambling [35], and probabilistic decision making [39], under a specific task condition it could still degrade performance (e.g., musicians train for many years to attain motor consistency and precision) [36]. Thus, the presence of stochastic noise in the brain should impose an inverted-U relationship between trial-to-trial brain activity and performance. Using electrocorticography (ECoG) in patients undergoing invasive brain monitoring for neurosurgical treatment, we found strong evidence for such a phenomenon in the human brain.

## Results

**Figure 1:**
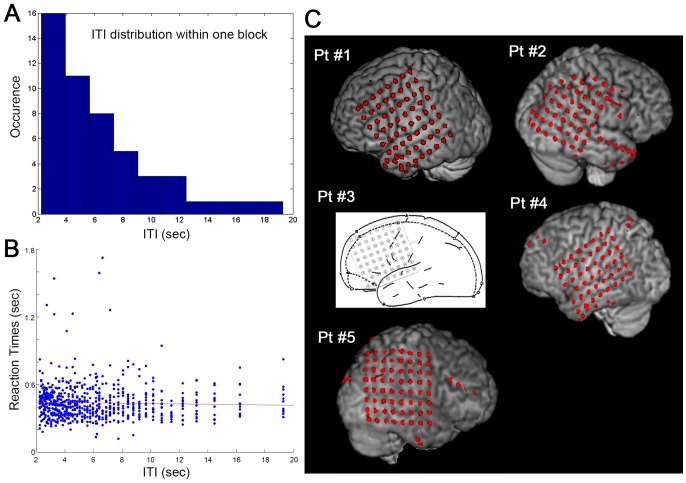
Task design, behavioral data and electrode coverage. (**A**) The distribution of inter-trial intervals (ITIs) in one task block containing 50 trials. This distribution is identical across blocks. (**B**) A scatter plot of reaction times (RT) against ITI across all hit trials in all subjects over contralateral blocks. There was no dependence of RT on ITI (P>0.1, Spearman rank correlation). The red line indicates the best linear regression fit. (**C**) Electrode locations in each subject overlaid on the pial surface reconstructed from the subject's own anatomical MRI. All intracranial electrodes are shown, including electrodes excluded due to signal quality issues or from the Laplacian montage derivation (those on the electrode strips or on the edge of the grid). For Pt #3, the clinical CT scan was not acquired, thus electrode locations could not be determined in relation to the MRI and the presurgical planning diagram is shown instead.

Five patients undergoing invasive brain monitoring for treatment of epilepsy performed a visuomotor detection task (for details see Materials and Methods). They fixated on a white cross in the center of a black screen that occasionally changed to dark grey for 250 milliseconds at times unpredictable to the subject (inter-trial intervals (ITI) ranged from 2 to 19.04 sec, Fig. 1A), and were instructed to press a button as quickly as they detected the cue. Each subject completed 6∼8 blocks of visual detection task, alternating between the use of left and right index finger for button press. Overall, 149∼200 trials were obtained in each subject under contralateral or ipsilateral finger use (see Table 1). Their reaction times (RTs) did not depend on ITI (Fig. 1B), suggesting a flat hazard rate, i.e., subjects were unable to predict the upcoming stimulus.

**Table 1:** Demographic, clinical and data collection information.

Pt#	Age	Gender	Handed-ness	Seizure focus	No. of grid electrodes	No. of Laplacian electrodes
1	45	F	R	L medial temporal	64	36
2	59	F	L	R inferior lateral parietal	62	32
3	58	F	R	L frontal	56	25
4	36	M	L	L medial temporal	48	24
5	19	M	R	R lateral temporal	64	36

Contra-lateral data					
Pt#	Total No. of trials	No. of hit trials	No. of miss trials	Hit rate (%)	Median RT (ms)
1	149	83	66	55.7	460
2	150	138	12	92.0	350
3	200	195	5	97.5	410
4	150	137	13	91.3	510
5	150	142	8	94.7	340

Ipsi-lateral data					
Pt#	Total No. of trials	No. of hit trials	No. of miss trials	Hit rate (%)	Median RT (ms)
1	150	99	51	66	415
2	150	143	7	95.3	360
3	200	192	8	96	418
4	150	129	21	86	510
5	150	145	5	96.7	365

In total, 294 electrodes with good signal quality and no interictal spike-wave discharges were recorded across the five subjects. Demographic, clinical and data collection information in each subject is included in Table 1, and the electrode locations are shown in Fig. 1C. In order to focus on local brain activity underneath each electrode, all ECoG data were transformed into a Laplacian montage (see Materials and Methods). This resulted in 153 Laplacian electrodes in total. The Laplacian montage approximates transcortical recording (i.e., with the recording electrode on the cortical surface and the reference electrode in the underlying white matter), under which surface negativity in general indexes increased excitability, and surface positivity decreased excitability [40], [41].

### Trial-to-Trial Variability Reduces after Stimulus Onset

Recent studies have reported that trial-to-trial variability decreases after stimulus onset in neuronal spiking in primates and rodents [23]–[25], [28], [29], [42] as well as fMRI signals from humans [30], suggesting that the brain settles into a more confined state space under task stimulation. Moreover, variability reduction can be decoupled from trial-averaged activity in both stimulus modulation [23], [25], [30] and correlation with behavior [28], [30], predicting a U- or inverted-U relationship between trial-to-trial brain activity and behavioral performance. To investigate whether variability reduction might also be observed in ECoG recordings, we first characterized the across-trial mean (similar to ERP) and variability time courses for each electrode.

**Figure 2:**
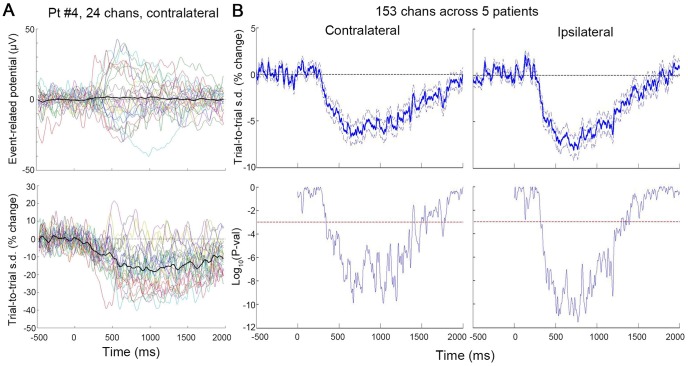
Reduction of trial-to-trial variability following stimulus onset. (**A**) Averaged ERPs (top) and trial-to-trial variability time courses (bottom) for all 24 Laplacian electrodes from Pt #4 (contralateral data). The variability time course was computed as standard deviation (s.d.) across trials, normalized to the mean of the pre-stimulus period (−500∼0 ms) and expressed in %change unit. Thick black traces denote the average across 24 electrodes. (**B**) Top: Trial-to-trial variability time course averaged across all 153 Laplacian electrodes in five subjects. Dashed lines depict mean±SEM. Bottom: Significance of the variability time course, assessed by a one-sample t-test across 153 electrodes against the null hypothesis of no change from baseline. The left column is obtained using contralateral data, and the right column using ipsilateral data. Red dashed lines indicate significance level of P = 0.001.

We first analyzed the contralateral data (i.e., the index finger contralateral to the electrode grid was used for motor output). Across 153 electrodes in five subjects, 76 electrodes showed net negative deflections and 77 showed net positive deflections in the trial-averaged activity (assessed by the integral in a 0∼1500 ms post-stimulus window). A majority of the electrodes (N = 104) exhibited reduction of trial-to-trial variability in the post-stimulus period (assessed by the integral in a 0∼1500 ms post-stimulus window). Fig. 2A shows the averaged ERP and trial-to-trial variability time courses for 24 Laplacian electrodes from a representative subject. Pooling across all 153 electrodes from five subjects, we observed a dramatic reduction of across-trial variability in the post-stimulus period that reached the minimum at 646 ms and gradually recovered to baseline at around 2 sec following the stimulus (Fig. 2B, left column). In single electrodes, the reduction of variability was as much as 57.5%.

Strikingly, similar variability reduction was observed when the ipsilateral hand was used for motor output (Fig. 2B, right column). Across 153 electrodes, the magnitude of variability reduction was slightly larger than when the contralateral hand was used (Fig. 2B). This result is consistent with previous findings showing that the ipsilateral hemisphere can exhibit variability reduction even without a change in the trial-averaged activity [25], [30] and that the magnitude of variability reduction in the ipsilateral cortex can exceed that in the contralateral cortex (see Fig. 6 in Ref [30]).

### Relationship between the 1^st^ Principal Component and Hit Rate

**Figure 3:**
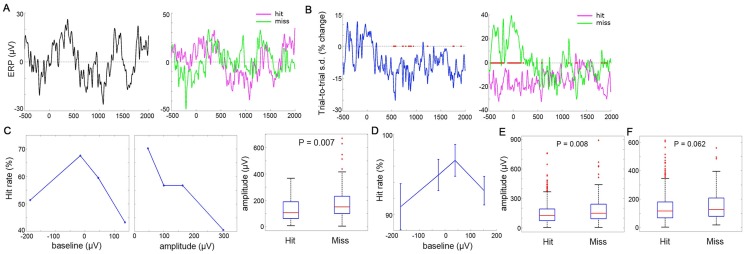
Inverted-U relationship between ECoG activity (the first PC) and hit rate. (**A–C**) Results from the first PC in Pt #1 (contralateral data). (**A**) Left: Averaged ERP across all trials. Right: Averaged ERP for hit and miss trials separately. (**B**) Left: Across-trial variability time course; red dots: P<0.005 (F-test, compared against pre-stimulus period). Right: Across-trial variability time course (normalized to the pre-stimulus mean computed across all trials) for hit (N = 83) and miss (N = 66) trials separately; red dots: P<0.005 (F-test, hit vs. miss trials). (**C**) Left: Hit rate as a function of raw ECoG activity at stimulus onset. Middle: Hit rate as a function of rectified ECoG signal amplitude at stimulus onset. Right: ECoG signal amplitude at stimulus onset for hit vs. miss trials (P = 0.007, Wilcoxon rank-sum test). Red line and the edges of the box denote median, 25^th^ and 75^th^ percentiles respectively. The whiskers extend to the range for data not considered outliers and the crosses indicate the outliers. (**D**) Hit rate as a function of ECoG activity (from the first PC) at stimulus onset, averaged across Patients #2–5 (contralateral data). (**E**) ECoG signal amplitude (from the first PC in each subject, contralateral data) at stimulus onset for hit vs. miss trials (P = 0.008, Wilcoxon rank-sum test). Data were pooled across all five subjects. (**F**) Same as **E**, except using ipsilateral data across 5 subjects. Hit vs. miss: P = 0.062 (Wilcoxon rank-sum test).

To test the Inverted-U hypothesis in relation to hit rate (using contralateral data), we first focused on a subject that had a sufficient number of miss trials to be analyzed individually (Patient #1, hit rate 55.7%, see Table 1). To reduce dimensionality, we applied PCA [43], [44] to data from all 36 electrodes, and extracted the first principal component (PC) accounting for the largest amount of variance. Trial-to-trial variability of this PC decreased in a sustained manner following stimulus onset (Fig. 3B, left), despite a non-significant averaged ERP (Fig. 3A, left).

We computed the across-trial variability time courses for hit and miss trials separately, and found significantly smaller variability in hit than miss trials around and *before* the stimulus onset (Fig. 3B, right). By contrast, the averaged ERP was indistinguishable between hit and miss trials (Fig. 3A, right). Consistent with earlier findings, these data suggest that behaviorally relevant information can be encoded solely within the across-trial variability of brain activity but not the across-trial mean [28], [30]. Smaller variability in hit than miss trials, without a difference in the trial-averaged activity, is consistent with an inverted-U relationship between trial-to-trial ECoG activity and hit rate.

To directly test the inverted-U relationship between ECoG activity and hit rate, we binned all trials into quartiles according to the activity of the first PC at stimulus onset, and calculated the hit rate for each quartile separately. We found that the relationship between hit rate and ECoG activity indeed followed an inverted-U function, such that both very low and very high ECoG activity at stimulus onset foreshadowed more misses (Fig. 3C, left). When the ECoG activity was close to the across-trial mean, hit rate was as much as 67.6%; whereas when it was most negative or most positive, hit rate dropped to 51.4% and 43.2%, respectively. An inverted-U relationship between trial-to-trial ECoG activity and hit rate should also manifest as a negative monotonic relationship between the rectified amplitude of ECoG activity and hit rate, as activity closer to the mean is associated with smaller amplitude (note that this relationship applies to broadband signals but not narrow-band oscillations; see Fig. S1). We thus extracted the instantaneous ECoG signal amplitude at stimulus onset via Hilbert transform (throughout the article, “amplitude” refers to rectified amplitude). By binning all trials into four groups according to amplitude, we indeed uncovered a negative monotonic dependence between hit rate and amplitude: Hit rate was 70.3% in the lowest amplitude bin, and it dropped to 40.5% in the highest amplitude bin (Fig. 3C, middle). Comparing hit with miss trials directly, we found that ECoG signal amplitude at stimulus onset was significantly smaller in hit than miss trials (Fig. 3C, right; P = 0.007, Wilcoxon rank-sum test).

Across the remaining four subjects, there was a similar inverted-U relationship between ECoG activity (the first PC extracted from each subject's data) at stimulus onset and hit rate (Fig. 3D, quartile binning within each subject). Combining data across all five subjects, the amplitude of the first PC at stimulus onset was significantly smaller in hit compared to miss trials (Fig. 3E; P = 0.008, Wilcoxon rank-sum test). These results suggest that the first PC extracted from the ECoG data shows an inverted-U relationship with hit rate, such that both very low and very high activity levels at stimulus onset predict more misses.

Lastly, we examined whether the inverted-U relationship between ECoG activity and hit rate might also exist when the ipsilateral hand was used for motor output. Again, PCA was applied to each subject's data to extract the first PC. Across five subjects, the amplitude of the first PC did not significantly differentiate between hit and miss trials, although there was a trend effect (Fig. 3F, P = 0.062, Wilcoxon rank-sum test).

### Analysis Combining across PCs

In order to generalize the above findings beyond the first PC, we applied PCA to each subject's data, sorted the PCs by the amount of variance they explained in descending order, and extracted the first 5 PCs in each subject. We sought to test whether the population activity reflected across multiple PCs might also exhibit an inverted-U relationship with behavior; we further assessed the dependence of such a relationship on the number of PCs included. To this end, we first computed the across-trial mean time course for each PC. We then obtained the distance (i.e., absolute difference) between its activity in each trial and its across-trial mean, and averaged this distance across the chosen number of PCs to obtain a “summary” distance time course for each trial – *D(t)* (for details see **Distance-to-Mean Analysis** in Materials and Methods). The Inverted-U hypothesis suggests that in a given trial, the farther the population activity is from the across-trial mean (i.e., the larger the *D(t)*), the worse the behavioral performance.

**Figure 4:**
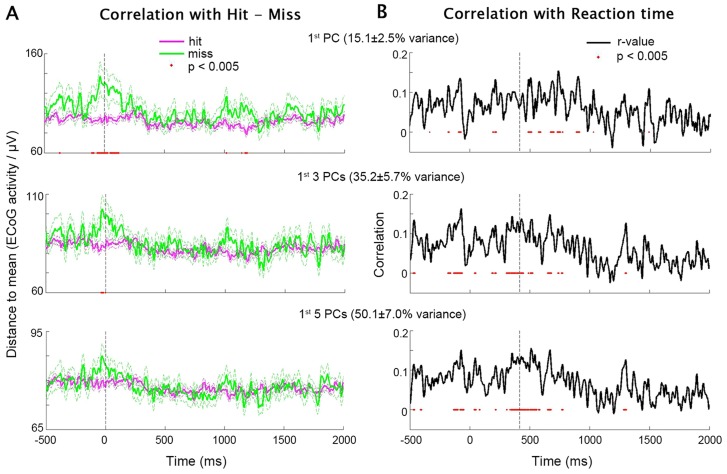
PCA test of the Inverted-U hypothesis using contralateral data. (**A**) *D(t)* combined across the first one (top), three (middle) and five (bottom) PCs in each subject, averaged for hit and miss trials separately (data from all subjects were included). Flanking dashed lines depict mean±SEM. Red dots: P<0.005 for hit vs. miss trials, two-sample t-test. Vertical dashed line indicates stimulus onset. (**B**) Time courses of Pearson correlation coefficient between *D(t)* and RT across all hit trials (including data from all subjects). *D(t)* was combined across the first one (top), three (middle) and five (bottom) PCs in each subject. Red dots: P<0.005 for significant *D(t)*-RT correlation. Vertical dashed line indicates the time of median RT across all subjects.

We first investigated the contralateral data. The top one, three or five PCs from each subject were included in the analysis, which accounted for 15.1±7.0% (mean±s.d. across subjects), 35.2±5.7% and 50.1±7.0% of total variance respectively. Pooling data across all subjects, *D(t)* was smaller in hit trials than miss trials around stimulus onset (t = 0 ms), regardless of the number of PCs included (Fig. 4A). Hence, at stimulus onset, activity closer to the across-trial mean predicted a higher hit rate. Interestingly, increasing the number of PCs included in the analysis from 1 to 5 progressively decreased the strength of this relationship (Fig. 4A), implying that the inverted-U relationship with hit rate was likely localized to a subset of the electrodes, a topic that we shall return to later.

Next, we computed the correlation between *D(t)* at each time point and RT across all hit trials from all subjects. This analysis revealed a positive correlation between RT and *D(t)* around the time of behavioral responses (Fig. 4B), suggesting that in a given trial, the closer the ECoG activity is to the across-trial mean, the faster the reaction time. Contrary to the hit-vs.-miss analysis (Fig. 4A), this effect was slightly stronger when more PCs were included in the analysis (Fig. 4B), indicating that the inverted-U relationship with RT was relatively distributed across electrodes. To ensure that the correlation between *D(t)* and RT was driven by trial-to-trial variability but not inter-subject differences, we plotted *D(t)* against RT across all trials from all subjects, and confirmed that the distributions for different subjects were largely overlapping (see Fig. S2).

**Figure 5:**
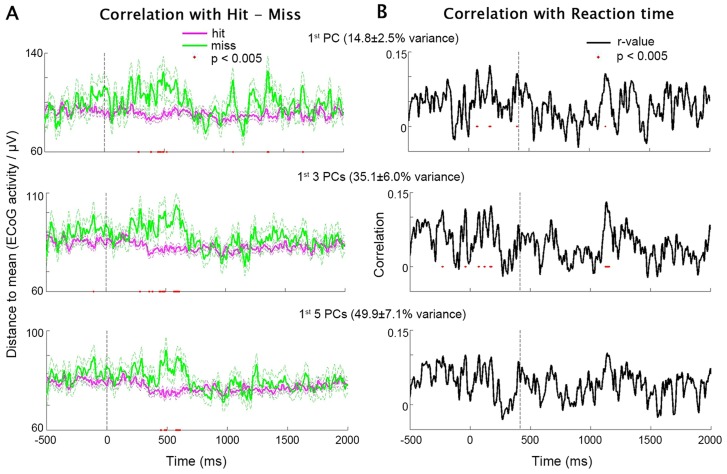
PCA test of the Inverted-U hypothesis using ipsilateral data. (**A** and **B**) same as in Figure 4, except results were obtained using ipsilateral data.

Results from similar analyses applied to ipsilateral data are shown in Fig. 5. The top one, three or five PCs from each subject accounted for 14.8±2.5% (mean±s.d. across subjects), 35.1±6.0% and 49.9±7.1% of total variance respectively. Interestingly, the difference in *D(t)* between hit and miss trials was more pronounced around behavioral responses in the ipsilateral data (Fig. 5A), as opposed to being around the stimulus onset in the contralateral data (Fig. 4A). In addition, the correlation between *D(t)* and RT was less robust and more sporadic in time in the ipsilateral data (Fig. 5B) as compared with the contralateral data (Fig. 4B).

### Electrode-by-Electrode Analysis

The above results demonstrate the existence of an inverted-U relationship between trial-to-trial brain activity and behavioral performance at the principal-component level. An important remaining question regards the spatial localizations of the inverted-U relationship vis-à-vis the classical monotonic brain-behavior relationship (e.g., [10], [18]). To address this question, we performed an electrode-by-electrode analysis on the contralateral data. Given the above results (Fig. 4), for hit-vs.-miss analysis we focused on ECoG activity at stimulus onset; for RT analysis we focused on ECoG activity around the behavioral responses.

**Figure 6:**
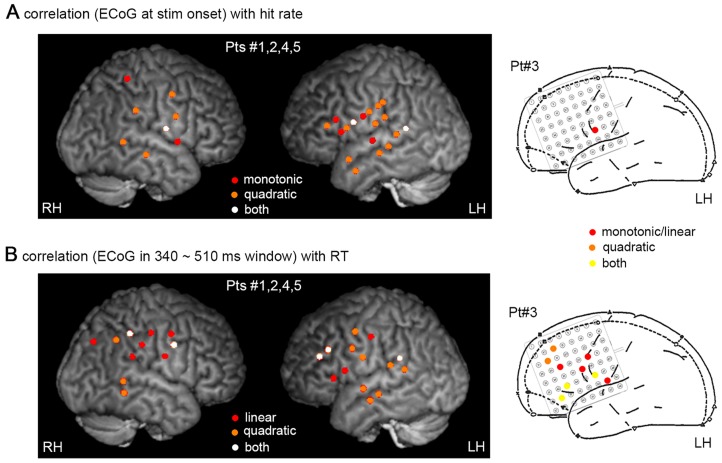
Electrode-by-electrode analysis (contralateral data). (**A**) Electrodes demonstrating a monotonic (red) or quadratic (orange) relationship (P<0.05) between ECoG activity at stimulus onset and hit rate. Electrodes with both relationships are shown in white. (**B**) Electrodes demonstrating a linear (red) or quadratic (orange) relationship (P<0.05) between ECoG activity around behavioral responses (averaged in a 340∼510 ms post-stimulus window) and RT. Electrodes with both relationships are shown in white for Pts #1,2,4,5 (left) and yellow for Pt #3 (right). Because CT data was not available for Pt #3, precise electrode localization could not be performed and presurgical planning diagram was used instead. LH: left hemisphere; RH: right hemisphere.

To identify a monotonic relationship between ECoG activity and hit rate, we directly compared the ECoG signal value at stimulus onset between hit and miss trials. To identify a U- or inverted-U- (i.e., quadratic) relationship between ECoG activity and hit rate, we compared the amplitude of the ECoG signal at stimulus onset between hit and miss trials (as in Fig. 3E). Larger amplitude in miss than hit trials indicates an inverted-U relationship between trial-to-trial ECoG activity and hit rate (see Fig. 3C). Across 153 electrodes in five subjects, 10 electrodes showed a significant monotonic relationship, which was not significant at the population level (population-level P = 0.09, binomial statistics [30]), with exactly half of them having higher activity in hit than miss trials. Twenty electrodes showed a significant quadratic relationship to hit rate (population-level P = 5.8e-5, binomial statistics), with 75% of them having larger amplitude in miss than hit trials (i.e., an inverted-U relationship between ECoG activity and hit rate). Three electrodes demonstrated both a significant monotonic and a significant quadratic relationship with hit rate. The spatial localizations of electrodes showing monotonic vs. quadratic relationships and their overlaps are shown in Fig. 6A.

To assess the relationship between ECoG activity and RT, for each electrode we averaged the ECoG activity within a 340∼510 ms post-stimulus window, as the median RT ranged from 340 to 510 ms across subjects (see Table 1). We then characterized both linear and quadratic relationships between the ECoG activity and RT across trials. In total, 21 electrodes showed a linear correlation between ECoG activity and RT (*R*T* = βx+c*, where *x* is the ECoG signal value; population-level P = 1.9e-5, binomial statistics), with 57% of them being a positive correlation. Twenty-one electrodes showed a significant quadratic relationship (*R*T* = αx^2^+βx+c*; population-level P = 1.9e-5, binomial statistics). The coefficient of the quadratic term (*α*) is positive in 20 out of 21 (i.e., 95.2%) electrodes , suggesting that both very low and very high ECoG activity levels were associated with slower RTs (i.e., an inverted-U relationship between ECoG activity and response speed). The spatial localizations of electrodes exhibiting linear vs. quadratic relationship with RT and their overlaps (N = 8) are shown in Fig. 6B.

**Figure 7:**
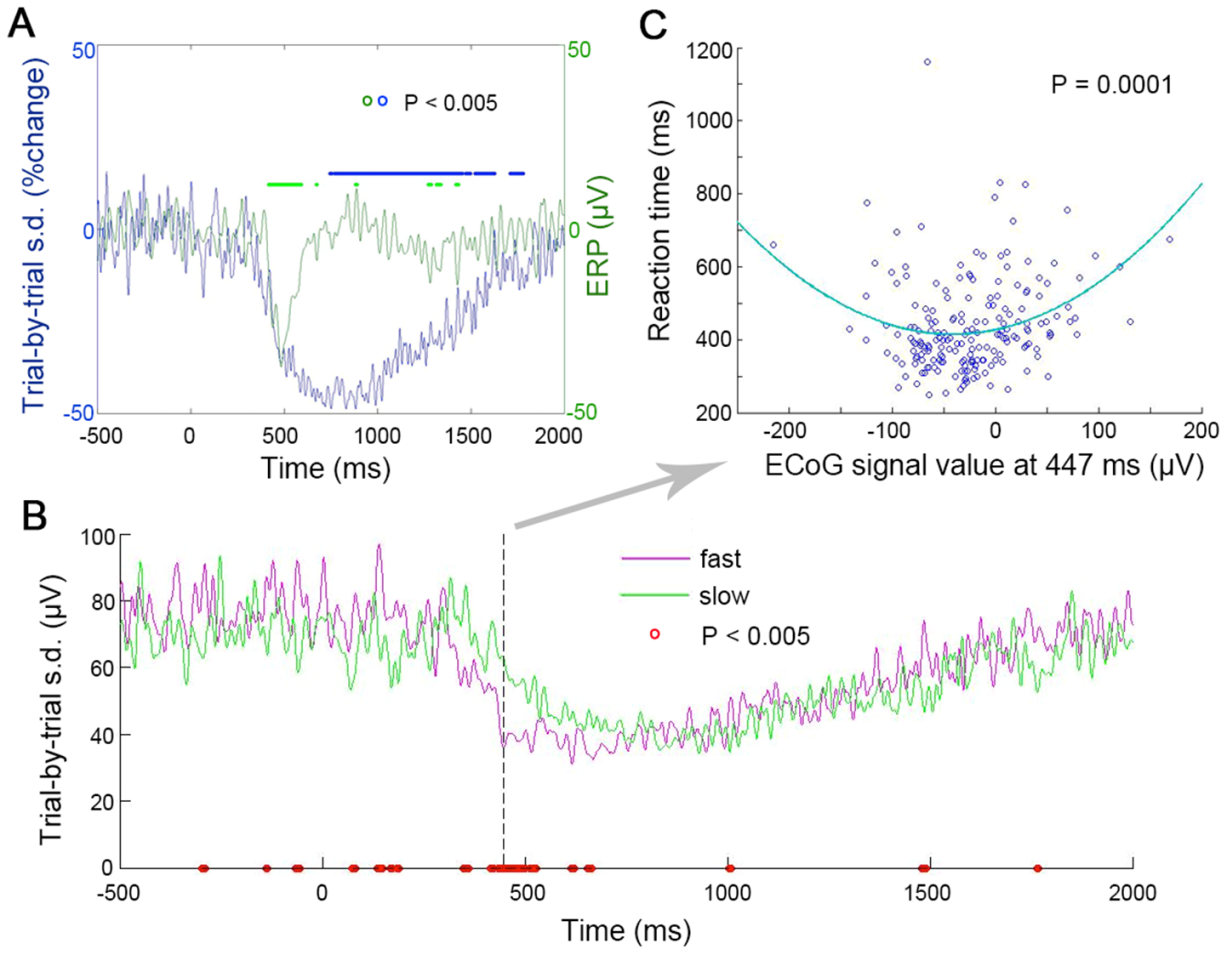
Inverted-U relationship between ECoG activity and response speed in an example electrode (from Pt #3, contralateral data). (**A**) Averaged ERP (green) and across-trial variability (blue) time courses. Green and blue dots indicate P<0.005 compared to the pre-stimulus period for ERP and variability respectively. (**B**) Across-trial variability time courses for fast and slow trials separately. Fast and slow trials were defined by a median-split on RT across all hit trials (N = 195). Red circles: P<0.005 for variability between fast and slow trials (two-sample F-test for variance). (**C**) Scatter plot of ECoG signal value at 447 ms against RT across all hit trials. Blue line indicates the best-fit quadratic function (P = 0.0001).

Overall, electrodes demonstrating monotonic and quadratic brain-behavior relationships tended to form separate but adjacent clusters with limited overlap between them (Fig. 6). Detailed characterizations of an example electrode showing a quadratic relationship with RT are shown in Fig. 7. Its across-trial variability time course showed sustained reduction following stimulus onset, despite a transient change in the averaged ERP (Fig. 7A). We separated all hit trials in this subject (N = 195) into two groups via a median-split on RT, and found significantly smaller trial-to-trial variability in this electrode for fast compared with slow trials at around 400∼500 ms (Fig. 7B). The difference in variability between fast and slow trials was most pronounced at 447 ms following stimulus onset (P = 1.1e-7, two-sample F-test). A scatter plot of the ECoG activity at 447 ms against RT across all hit trials is shown in Fig. 6C, which can be described by a U-shaped function (P = 0.0001). Importantly, at slower RTs, the distribution of the ECoG activity is wider, encompassing low, medium and high values; by contrast, faster RTs are accompanied by a narrow distribution of ECoG activity around medium values. This is consistent with the idea that a higher level of stochastic noise, which scatters the ECoG activity across a wider range, accompanies slower reaction times.

### Analysis Combining across Electrodes

So far we have demonstrated an inverted-U relationship between task performance and ECoG activity at the principal-component and single-electrode levels. These results suggest that in a given trial, the closer the ECoG activity is to the across-trial mean, the better the behavioral performance. Lastly, we sought to test the Inverted-U hypothesis by combining information across all electrodes. Because task processing requires coordinated actions from distributed areas of the brain [45]–[47], we reasoned that if the system as a whole is farther away from its targeted trajectory (approximated here roughly by the across-trial mean activity), behavioral performance should be compromised.

To this end, we computed the distance-to-mean measure *D_i_(t)* for each electrode instead of each PC, using the contralateral data. Then, for both analyses on hit rate and RT, we obtained a “summary” distance time course *D(t)* for each trial by averaging *D_i_(t)* across three electrode groups separately: i) all electrodes showing a significant quadratic relationship with hit rate or RT (see Fig. 6 A or B); ii) all other non-significant electrodes; and iii) all electrodes. For the analysis on hit rate, we tested the difference of *D(t)* between hit and miss trials; for the analysis on RT, we assessed the linear correlation of *D(t)* and RT across all hit trials (as in Figs. 4 & 5).

**Figure 8:**
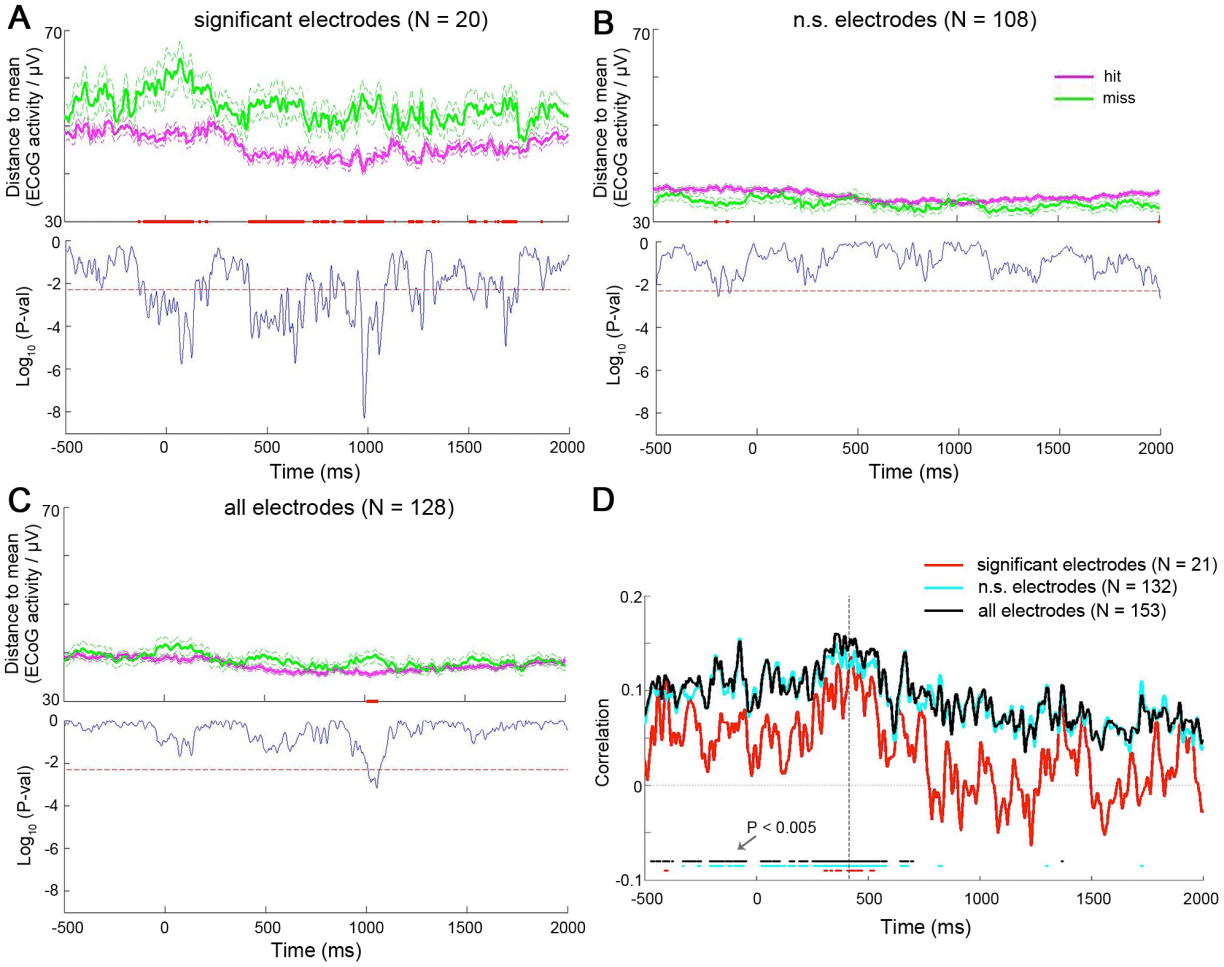
Analysis combining all electrodes (contralateral data). (**A**) Top: For each subject, *D(t)* was computed by combining across all significant electrodes in the electrode-based analysis (orange and white electrodes in Fig. 5A), and then averaged for hit and miss trials separately. Flanking dashed lines depict mean±SEM. Red dots: P<0.005 for hit vs. miss trials, two-sample t-test. Bottom: the P-value time course of the two-sample t-test comparing *D(t)* between hit and miss trials. Dashed red line indicates significance level of P = 0.005. (**B**) As in (A), except that *D(t)* was computed by combining across all remaining non-significant electrodes not included in (A). (**C**) As in (A), except that *D(t)* was computed by combining across all electrodes. For (A–C), all subjects except Pt #3 were included, because Pt #3 did not have any electrode showing a significant quadratic ECoG-hit rate relationship (see Fig. 5A). (**D**) Pearson correlation coefficient between *D(t)* and RT across all hit trials (pooled across all five subjects). *D(t)* was combined across all significant electrodes from the electrode-based analysis (orange/white/yellow electrodes in Fig. 6B) (red line), all remaining non-significant electrodes (blue line) and all (black line) electrodes. Dots at the bottom: P<0.005 for significant *D(t)*-RT correlation, with *D(t)* computed using all significant (red), all non-significant (blue) or all (black) electrodes. Vertical dashed line indicates the time of median RT across all subjects.

Combining information across all significant electrodes, we found that *D(t)* differentiated between hit and miss trials throughout the trial (Fig. 8A). By contrast, the difference between hit and miss trials was much diminished when information was combined across all non-significant electrodes (Fig. 8B) or all electrodes (Fig. 8C). This is consistent with our earlier result (Fig. 4A) implicating that the inverted-U relationship between ECoG activity and hit rate was relatively localized to a subset of electrodes.

From the analysis on RT, we found that combining information across all significant electrodes yielded a significant correlation between RT and *D(t)* mostly around behavioral responses (Fig. 8D, red). Remarkably, combining information across non-significant electrodes yielded stronger correlation throughout the trial (Fig. 8D, blue), as did combining information across all electrodes (Fig. 8D, black). The *D(t)*-RT correlation was present as early as 500 ms before the stimulus onset when information from all electrodes were combined. This result confirms the earlier impression (Fig. 4B) that the inverted-U relationship between ECoG activity and response speed was relatively distributed across electrodes.

## Discussion

In summary, consistent with earlier studies [7]–[9], the present results provide strong evidence for an inverted-U relationship between trial-to-trial brain activity and behavioral performance in the human brain, such that moderate activity predicts better behavioral performance (more hits and shorter RTs), whereas both very low and very high activity levels are associated with degraded performance (more misses and longer RTs). While these previous studies have focused on the amplitude of alpha and mu oscillations in sensory cortices, the present study extends the inverted-U relationship to the raw fluctuations of field potentials (similar to single-trial ERPs) outside the sensory cortex. Specifically, when the contralateral hand was used for motor output, we found that the inverted-U relationship between trial-to-trial ECoG activity and hit rate was most pronounced around the stimulus onset (Fig. 4A) and was relatively localized to a subset of electrodes (Fig. 8 A–C). By contrast, the inverted-U relationship between ECoG activity and response speed was strongest around behavioral responses (Fig. 4B) and was more distributed across all electrodes (Fig. 8D). Interestingly, when the ipsilateral hand was used for motor output instead, the inverted-U relationship between ECoG activity and hit rate was most pronounced around behavioral responses (Fig. 5A), and that between ECoG activity and response speed was less robust and more sporadic in time (Fig. 5B).

The Laplacian montage of ECoG approximates transcortical recording, such that increased cortical excitability manifests as more negative ECoG signals. Thus, the inverted-U relationship between trial-to-trial ECoG activity and behavioral performance suggests that both very low and very high cortical activity, manifesting as most positive and most negative ECoG signals respectively, are associated with compromised performance, while intermediate activity levels are associated with better performance. These findings support the notion that a higher level of stochastic noise, which scatters the brain activity across a wider range, is associated with degraded performance under a specific task condition. Importantly, such an effect is not contradictory to a potentially beneficial role of stochastic noise during development or evolution [35], [48]. For example, young adulthood is associated with larger brain variability as compared with both childhood and aging, and at the same time more accurate and consistent behavioral responses [49]–[51]. We think that these observations are complementary instead of contradictory to the present results, as both a wider dynamic range at rest and the ability to quickly settle into the desired state during task should be associated with better function. Thus, across developmental stages, larger brain variability is an index of a wider dynamic range and a greater repertoire of potential brain states [48]. On the other hand, during task processing, the ability of the brain to quickly settle into a particular task state is highly desirable and predictive of better performance. Indeed, post-stimulus brain responses appear to be more variable in patients with autism [27] and schizophrenia [52].

An inverted-U brain-behavior relationship is consistent with the present (Figs. 3B & 7B) and earlier [28], [30] findings showing that better behavioral performance can be accompanied by smaller trial-to-trial variability, even without any correlation with trial-averaged activity. An inverted-U relationship has also been reported between trial-to-trial neuronal firing rate and movement speed in the macaque premotor cortex [29]. In addition to the different recording modalities and species, the present results extend this previous finding in several directions by showing: i) the inverted-U relationship is present in both post-stimulus brain responses and pre-stimulus ongoing activity up to 500 ms before the stimulus onset; ii) the inverted-U relationship had largely separate spatial localizations from the monotonic brain-behavior relationship (Fig. 6); and iii) the spatiotemporal patterns of the inverted-U relationship differ between performance metrics (hit rate vs. RT) and motor output (contralateral vs. ipsilateral) (Figs. 4, 5 & 8), suggesting functional specificity.

The difference in spatial localization for the inverted-U vs. monotonic brain-behavior relationships is consistent with our earlier observation that variability-based analysis results follow a different spatial distribution from mean-based analysis results [30]. It is worth noting that the exact spatial localizations of the quadratic and monotonic brain-behavior relationships in our electrode-by-electrode analysis (Fig. 6) should not be over-interpreted. This is partly because the spatial localizations revealed by ECoG in neurosurgical patients are subject to limited electrode coverage (especially under Laplacian montage) as well as heterogeneous spatial sampling from subject to subject (Fig. 1C). More importantly, as revealed in our analysis on RT (Fig. 8D), there is a significant amount of behaviorally relevant information distributed among non-significant electrodes. Our PCA analysis also suggests that the inverted-U relationship with RT is distributed amongst the top PCs (Fig. 4B). These results are consistent with a recent fMRI study showing that with sufficient signal-to-noise ratio, over 95% of the brain is involved in a simple visual attention task [47]. Lastly, a limitation of the present study is that the primary (visual) sensory regions were not sampled by the ECoG electrodes (see Fig. 1C), hindering a direct comparison with earlier studies on this topic [7]–[9]. Nonetheless, our results in somatosensory/motor regions are consistent with earlier neurophysiological studies in the macaque [23], [29].

The traditional framework in neuroscience surmises that brain responses during task are the superposition of noise-like ongoing activity and task-evoked activity. This framework predicts variability increase after task onset [30] and a monotonic relationship between trial-to-trial brain activity and behavioral performance. Our findings of stimulus-induced variability reduction and an inverted-U brain-behavior relationship cannot be accommodated within this traditional framework. Instead, our results are readily embraced by attractor-network [53]–[55] and liquid-state machine [56]–[58] theories. These theories predict that as the network settles into a particular state or trajectory during task processing, across-trial variability decreases [55], [59]. The faster the system converges onto this state, the more similar its activity will be across trials, predicting a correlation between fast RT and smaller across-trial variability (see Fig. 7B herein and Ref [28]–[30]). Moreover, because the presence of stochastic noise scatters brain activity across a wider range than the targeted state, the closer the brain activity is to the targeted trajectory, the better the behavioral performance, consistent with the inverted-U relationship we observed. Notably, these ideas have close parallels to the “optimal-subspace hypothesis” developed in the context of motor preparation [60].

While the attractor-network framework provides potential explanations for our observation of an inverted-U relationship between post-stimulus brain activity and performance, what could account for the presence of such a relationship at or before the stimulus onset? One potential explanation for our results might be provided by the sampling-based Bayesian framework [61]. This idea proposes that the brain activity trajectory through the multidimensional state space samples different potential states; the distribution of the pre-stimulus samples constitutes the prior of its internal model, and that of the post-stimulus samples constitutes the posterior of the model. Accordingly, pre-stimulus activity closer to the across-trial mean would represent cortical states that are *a priori* more probable (assuming a unimodal distribution). Speculatively, this “most probable” state might also be the most “ready” state, and thus be associated with better performance. Another, not mutually exclusive, explanation lies in the framework of stochastic resonance – a nonlinear effect whereby an optimal level of noise facilitates the detection of a weak stimulus [62], [63]. This framework has been invoked previously to explain the inverted-U relationship between pre-stimulus amplitude of brain oscillations and task performance [7]. Since low-frequency activity phase modulates higher-frequency power [31], [32], such a mechanism might explain our results pertaining to the raw fluctuations of broadband signals as well. In addition, a theoretical model utilizing input-output nonlinearity to explain an inverted-U relationship between pre-stimulus oscillatory power and ERP has been proposed [8]. Needless to say, the exact mechanisms of the inverted-U brain-behavior relationship we uncovered in the pre-stimulus period await future investigations. In particular, elucidation of the interaction between higher-frequency brain oscillations and lower-frequency raw signal fluctuations (such as the slow-cortical potentials) [31], [32], [64], [65] in the context of inverted-U brain-behavior relationship might provide clues to the underlying mechanism.

In conclusion, our results are in line with recent theoretical frameworks suggesting that the brain is an active nonlinear dynamical system whose activity trajectory embodies information processing [48], [56], [57], [66]–[69]. Interestingly, the inverted-U and monotonic relationships between brain activity and behavioral performance were largely segregated in their spatial localizations (Fig. 6). While the presence of stochastic noise in the brain could impose an inverted-U relationship between brain activity and behavioral performance, it is tempting to speculate that the monotonic relationship might have a larger contribution from the deterministic source of brain variability. The underlying mechanisms of these two dissociable types of brain-behavior relationships should be an important topic for future experimental and theoretical studies.

## Materials and Methods

### Ethics Statement

All patients gave informed consent, after full explanation of the experiment, according to the procedures established by Washington University Institutional Review Board (Protocol: #06-1234, PI: John Zempel).

### Subjects

Five patients undergoing surgical treatment for intractable epilepsy participated in the study. All patients had complex partial seizures. To localize epileptogenic zones, patients underwent a craniotomy for subdural placement of electrode grids and strips followed by 1–2 weeks of continuous video and ECoG monitoring. The placement of the electrodes and the duration of monitoring were determined solely by clinical considerations. Exclusion criteria were: (1) widespread interictal spike-and-wave discharges; (2) age <15 years old; (3) severely impaired cognitive capability; (4) diffuse brain tissue abnormality, e.g., tuberous sclerosis, cerebral palsy; (5) limited electrode coverage. See Table 1 for demographic, clinical and data collection information. Other analyses on data from Patients #1–3, not relevant to the current topic, have been published in a previous paper [31].

### ECoG Data Acquisition

The electrode arrays (typically 8×8, 6×8 or 2×5) and strips (typically 1×6 or 1×8) consisted of platinum electrodes of 4-mm diameter (2.3 mm exposed) with a center-to-center distance of 10 mm between adjacent electrodes (AD-TECH Medical Instrument Corporation, Racine WI). ECoG signals were split and sent to both the clinical EEG system and a research EEG system (SynAmp^2^ RT, Neuroscan, DC-coupled recording). All data in the present study were from the research amplifier. Sampling frequency was 1000 Hz. Noisy electrodes and electrodes overlying pathologic tissue (including both the primary epileptogenic zone and areas showing active interictal discharges) were eliminated from all analyses.

### Task

Subjects fixated on a white cross in the center of a black screen; the cross occasionally changed to dark grey for 250 ms at times unpredictable to the subject. Inter-trial intervals (ITIs) ranged from 2 to 19.04 sec, randomly drawn from an Exponential distribution (Fig. 1A). Subjects were instructed to press a button as quickly as they detected the cue. Their force and reaction times (RTs) were recorded. Each task block contained 50 trials, lasting about 5 min. Subjects alternated between the use of left and right index fingers in different blocks. Each subject completed 6∼8 blocks in total. Task blocks using the finger contralateral and ipsilateral to the electrode grid (coverage was confined to one hemisphere in each patient) were analyzed separately. Unless otherwise noted, the reported results were computed using the contralateral blocks. The total number of trials completed by each subject ranged from 299 to 400 (see Table 1).

### Anatomical Magnetic Resonance Imaging (MRI) Acquisition

MRI was conducted at the Washington University Neuroimaging Laboratories either before admission or after discharge from the hospital. Patients were compensated for their time. Scanning was performed on a Siemens 3-T Trio MRI scanner. Anatomical images were acquired using a sagittal T1-weighted MP-RAGE sequence (TR = 2200 ms, TE = 2.34 ms, flip angle = 7°, inversion time = 1000 ms, 1×1×1 mm^3^ voxels). The MRI of each patient was co-registered to an atlas representative template, which was produced by mutual coregistration of MP-RAGE images obtained in 12 normal subjects and represented the Talairach coordinate system [70].

### Electrode Localization

Electrode localization followed procedures described previously [40]. Plain radiographs and computed tomography (CT) scans were acquired postoperatively with the subdural electrodes in place to define the electrode positions in relation to the skull. The CT images were co-registered to the subject's own anatomical MR image and then to the atlas-representative image. The Talairach coordinates of the center of each electrode were then determined using a custom-written automated procedure. Three-dimensional renderings of the pial surface were generated from atlas-transformed anatomical MR images using MRIcro (http://www.mccauslandcenter.sc.edu/mricro/mricro/). Because the CT scan was not acquired for Pt #3, precise electrode localization could not be performed and the clinical presurgical planning diagram was used instead.

### ECoG Data Preprocessing

In order to focus on brain activity directly underneath each electrode, we re-referenced the ECoG data to a Laplacian montage (similar as in Ref [40]). Under the Laplacian montage, the signal for each electrode is derived from the difference between this electrode and four surrounding electrodes that are nearest neighbors (1 cm apart from the center electrode). This necessitates the presence of four surrounding electrodes, so only electrodes on the grids (less the border rows) without an excluded electrode in the vicinity can be used as the center electrodes. The number of electrodes contributing to the Laplacian montage derivation (including center and reference electrodes) in each subject ranged from 48 to 64, and the final number of Laplacian electrodes ranged from 24 to 36 in each subject (see Table 1). Lastly, ECoG signals were filtered in 0.05∼50 Hz range with a 3^rd^-order acausal Butterworth filter offline, before any further analyses. The choice of the low-pass filter at 50 Hz was to avoid power line noise at 60 Hz. High-pass filter at 0.05 Hz was chosen in order to remove slow artifacts including electrode drift, but to retain as much of physiological signals in the low-frequency range as possible. It was an empirically determined value based on our recording set-up.

### Event-Related Potential (ERP) Analysis

In all analyses, time 0 indicates stimulus onset (the onset of crosshair dim). ERPs were obtained by averaging across trials in an epoch of −500∼2000 ms. No baseline correction was conducted in order to allow unbiased analyses of pre-stimulus activity in relation to behavioral performance. The significance of the ERP at single-electrode level was assessed by a paired t-test of post-stimulus activity at every time point against the pre-stimulus activity (averaged in a −500∼0 ms window) across trials. Difference of the ERP between hit and miss trials was assessed by a two-sample t-test at every time point.

### Across-Trial Variability Analysis

Across-trial variability time courses were computed for each electrode as the standard deviation (s.d.) of ECoG signal across trials at each time point from 500 ms before to 2 sec after the stimulus onset. The time courses were normalized to the pre-stimulus mean (averaged in a −500∼0 ms time window) and expressed in %change unit.

To assess significant post-stimulus increase or decrease of across-trial variability for a single electrode or principal component (PC), we used a two-sample F-test for variance. To increase robustness, the pre-stimulus activity was averaged within a window of −100∼0 ms, and the post-stimulus activity was averaged within a 100-ms-long window centered at the time point of interest. The F-test was conducted for pre-stimulus vs. post-stimulus activity across all trials, and was carried out for each post-stimulus time point from 50 to 1950 ms.

For hit vs. miss or fast vs. slow variability analyses, across-trial variability time courses were computed for each subgroup of trials (hit, miss, fast, slow). Difference in variability between two groups of trials was assessed by a two-sample F-test for variance. To increase robustness, the ECoG signal in each trial was averaged within a 100-ms-long window centered on the time point of interest. This test was carried out for every time point from −450 to 1950 ms.

### Principal Component Analysis (PCA)

For each subject, we computed the covariance matrix across all electrodes. The covariance matrix was computed on ECoG activity in the −500∼2000 ms window for each trial and averaged across all trials. Then PCA was applied to the averaged covariance matrix using pcacov function in Matlab (the Mathworks, Inc.). The coefficients from PCA were applied to the original ECoG signals in each trial to extract the principal components (PCs).

### Relationship between ECoG Activity and Hit Rate (PC-Level Analysis)

To investigate the relationship between trial-to-trial pre-stimulus ECoG activity and hit rate, we sorted all trials from each subject into four quartiles according to the pre-stimulus ECoG activity (see Table 1 for the total number of trials in each subject), and calculated the hit rate for each quartile separately. Approximately 37∼50 trials were present in each bin. Two metrics for defining the pre-stimulus activity were used: 1) the averaged ECoG activity in a pre-stimulus window of −200∼0 ms; and 2) the instantaneous ECoG activity value at stimulus onset. The two metrics gave similar results, thus we only report results using the instantaneous activity value here.

In addition, Hilbert transform was applied to ECoG activity in each trial to obtain the instantaneous amplitude signal. The amplitude at stimulus onset was analyzed in relation to hit rate. Two analyses were carried out: (i) All trials in each subject were sorted into quartiles according to the ECoG signal amplitude at stimulus onset, and then hit rate was computed for each quartile; (ii) The amplitude was directly compared between hit and miss trials via a Wilcoxon rank-sum test.

### Distance-to-Mean Analysis

To combine information across electrodes or principal components, we first computed the mean across trials for each electrode/PC:
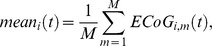
where *i* is the electrode/PC index (*i* = 1,2,… N), *t* ranges from −500 ms to 2000 ms, and *m* is the trial number (*m* = 1,2,… *M*). Then for each trial, we averaged the distance (i.e., absolute difference) to the mean across electrodes/PCs:
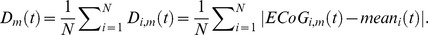
Thus, we obtained a *D(t)* time course for each trial in each subject. Next, we compared *D(t)* between hit and miss trials (combining data across all subjects) using a two-sample t-test at each time point *t*. A smaller *D(t)* in hit than miss trials suggests that activity closer to the mean is associated with a higher hit rate. For each time point *t*, we also calculated the linear correlation between *D(t)* and RT across all hit trials, again combining data across all subjects. A significant positive correlation indicates that the farther away the system is from the across-trial mean, the larger the RT (i.e., an inverted-U relationship between trial-to-trial ECoG activity and response speed).

### Electrode-Level Analysis

For each electrode, we investigated the dependence of hit rate on trial-to-trial ECoG activity at stimulus onset. A monotonic relationship was tested by a two-sample t-test on the ECoG activity value at stimulus onset between hit and miss trials. A quadratic relationship was tested by a Wilcoxon rank-sum test of the ECoG signal amplitude at stimulus onset between hit and miss trials. A smaller amplitude in hit as compared to miss trials means that hit rate was higher when ECoG activity was closer to the mean.

To evaluate the dependence of RT on trial-to-trial ECoG activity, we first averaged the ECoG activity in a post-stimulus 340∼510 ms time window (because the median RT in each subject ranged from 340 to 510 ms). Both linear (*R*T* = βx+c*) and quadratic (*R*T = *αx^2^+βx+c*) relationships were assessed between ECoG activity (*x* in the above equations) and RT across all hit trials, using corr and regstats functions in Matlab respectively. For a significant quadratic relationship, if the coefficient *α* is positive, that means RT is shorter when ECoG activity was closer to the across-trial mean (i.e., an inverted-U relationship between ECoG activity and response speed).

### Correction for Multiple Comparisons in the Time Domain

Several of our analyses relied on statistical tests on every time sample (Figs. 3A–B, 4, 5, 7A–B & 8). Thus, it is important to control for false positive rate due to the multiple comparisons carried out. Because adjacent time points in broadband ECoG activity are highly correlated [31], statistical tests on different time points are not independent. To account for temporal autocorrelation in the ECoG signal and derive the true degree of freedom in a 2.5-sec epoch, we employed Bartlett's theory [71]. The Bartlett correction factor (BCF) was calculated for every electrode as the integral of the squared lagged-autocorrelation function [72], [73]. The median of BCF across 153 electrodes for contralateral and ipsilateral data was 307.4 and 266.3, respectively. Since the task epoch contained 2501 time points, the upper limit for the number of independent tests was 2501/266.3 = 9.4. Hence, under Bonferroni correction, a P-value of 0.005 for the uncorrected test would fall in the range of P<0.05 after correction for multiple comparisons. We therefore thresholded all our results at a significance level of P<0.005 (uncorrected).

## Supporting Information

Figure S1
**Examples of Hilbert transform applied to broadband and narrowband signals to extract amplitude.** Related to Fig. 3 C–F. (**A**) Data from the first principal component (PC) in Patient #1 using randomly chosen trials. **Top**: The blue trace is the raw ECoG signal filtered in the 0.05∼50 Hz range (same band-pass filter as used in all data analyses). The red trace is the amplitude time series extracted by Hilbert transform. Notice that the time points in the raw signal that are close to 0 are associated with a smaller amplitude. Thus, amplitude extracted from the broadband data can supplement our time-domain analyses by converting an inverted-U relationship for raw ECoG activity (see Fig. 3C, left) to a negative monotonic relationship for amplitude (see Fig. 3C, middle), which is more amenable to statistical testing (see Fig. 3C, right). **Bottom**: Amplitude time series (red) extracted by Hilbert transform applied to the raw filtered time series in the 7∼13 Hz range (blue). Notice that for narrowband data, time points close to 0 are not associated with smaller amplitude. (**B**) Simulated fractional Brownian motion (fBm) [74], [75] with a power-law exponent of 1.7 (i.e., the power spectrum conforms to 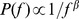), where β = 1.7). This choice of β is close to the power-law exponent of low-frequency ECoG activity [31]. Simulated fBm was filtered in the range 0.05∼50 Hz (top) and 9∼11 Hz (bottom) and the instantaneous amplitude was extracted via Hilbert transform. A narrower bandpass filter was used for the simulated fBm (9∼11 Hz) than for the ECoG data (7∼13 Hz) because the ECoG signal contained an alpha oscillation at ∼10 Hz, which was not present in the simulated fBm.(TIF)

Figure S2 **Correlation between RTs and** ***D(t)*** **across subjects.** Related to Fig. 4B (top). The first PC was extracted from each subject's data (using contralateral hand). Its distance-to-mean *D(t)* time course was computed for each trial and averaged within a post-stimulus 340∼510 ms window around the behavioral response, then plotted against RT across all hit trials in all subjects. Different colors indicate different subjects. The Pearson correlation coefficient and associated P-value computed across all subjects are indicated in the graph.
(TIF)

Table S1 **Talairach coordinates (mm) of electrodes showing a significant linear or quadratic relationship with hit rate (left column) or RT (right column), as well as electrodes showing both relationships with hit rate or RT.** Data from Patients #1, 2, 4 & 5 are included (see Fig. 6). Patient #3 is not included because the clinical CT scan was not obtained, thus the electrode locations in relation to MRI could not be determined.
(DOCX)
