# Supplementary figures and images for: Average Is Optimal: An Inverted-U Relationship between Trial-to-Trial Brain Activity and Behavioral Performance
Source: PLoS Comput Biol. 2013 Nov 7;9(11):e1003348. doi: 10.1371/journal.pcbi.1003348 (PMC3820514; doi:10.1371/journal.pcbi.1003348)

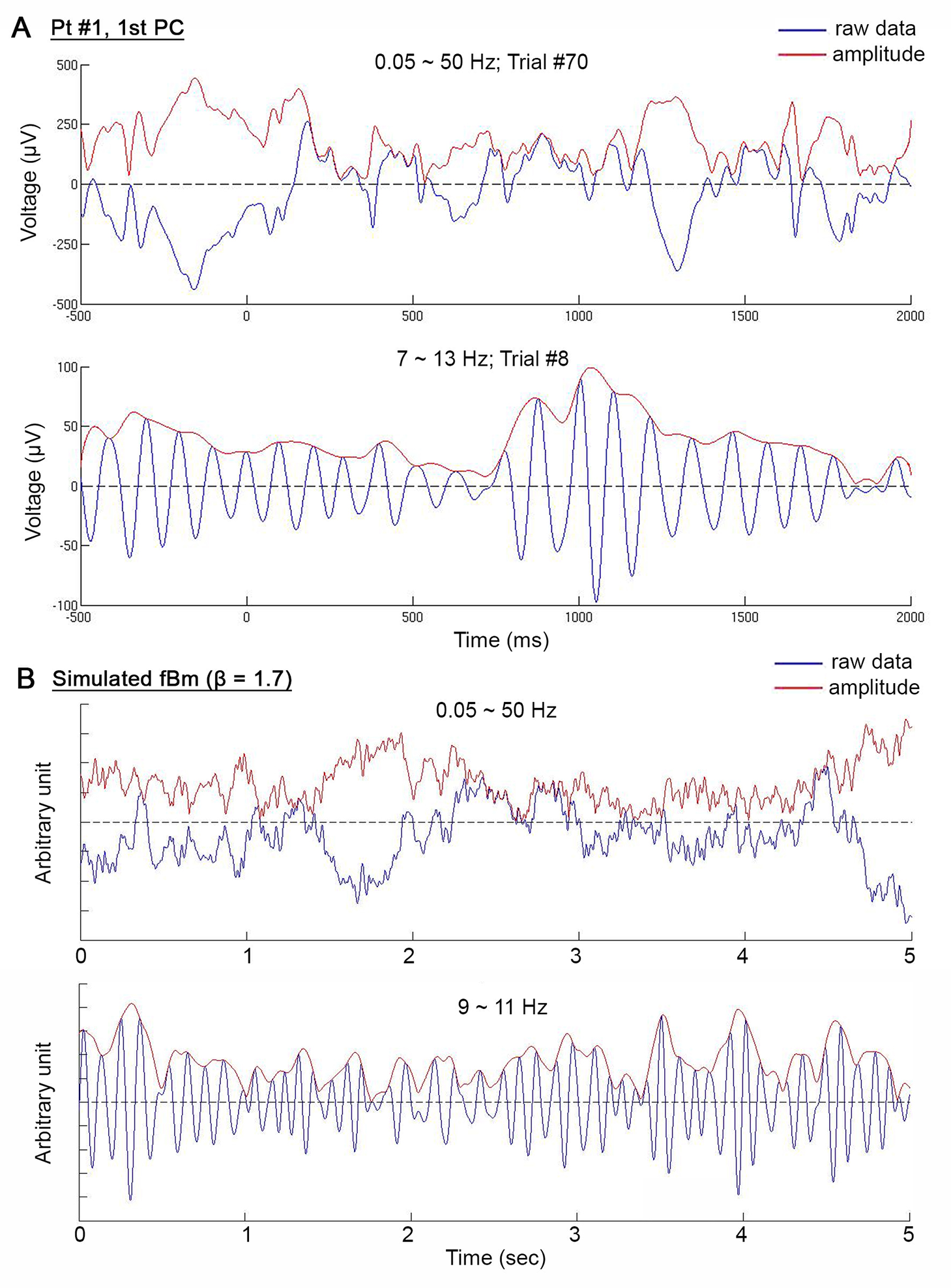

Supplement: Figure S1 — Examples of Hilbert transform applied to broadband and narrowband signals to extract amplitude. Related to Fig. 3 C–F. (A) Data from the first principal component (PC) in Patient #1 using randomly chosen trials. Top: The blue trace is the raw ECoG signal filtered in the 0.05∼50 Hz range (same band-pass filter as used in all data analyses). The red trace is the amplitude time series extracted by Hilbert transform. Notice that the time points in the raw signal that are close to 0 are associated with a smaller amplitude. Thus, amplitude extracted from the broadband data can supplement our time-domain analyses by converting an inverted-U relationship for raw ECoG activity (see Fig. 3C, left) to a negative monotonic relationship for amplitude (see Fig. 3C, middle), which is more amenable to statistical testing (see Fig. 3C, right). Bottom: Amplitude time series (red) extracted by Hilbert transform applied to the raw filtered time series in the 7∼13 Hz range (blue). Notice that for narrowband data, time points close to 0 are not associated with smaller amplitude. (B) Simulated fractional Brownian motion (fBm) [74], [75] with a power-law exponent of 1.7 (i.e., the power spectrum conforms to ), where β = 1.7). This choice of β is close to the power-law exponent of low-frequency ECoG activity [31]. Simulated fBm was filtered in the range 0.05∼50 Hz (top) and 9∼11 Hz (bottom) and the instantaneous amplitude was extracted via Hilbert transform. A narrower bandpass filter was used for the simulated fBm (9∼11 Hz) than for the ECoG data (7∼13 Hz) because the ECoG signal contained an alpha oscillation at ∼10 Hz, which was not present in the simulated fBm. (TIF) [file pcbi.1003348.s001.tif]

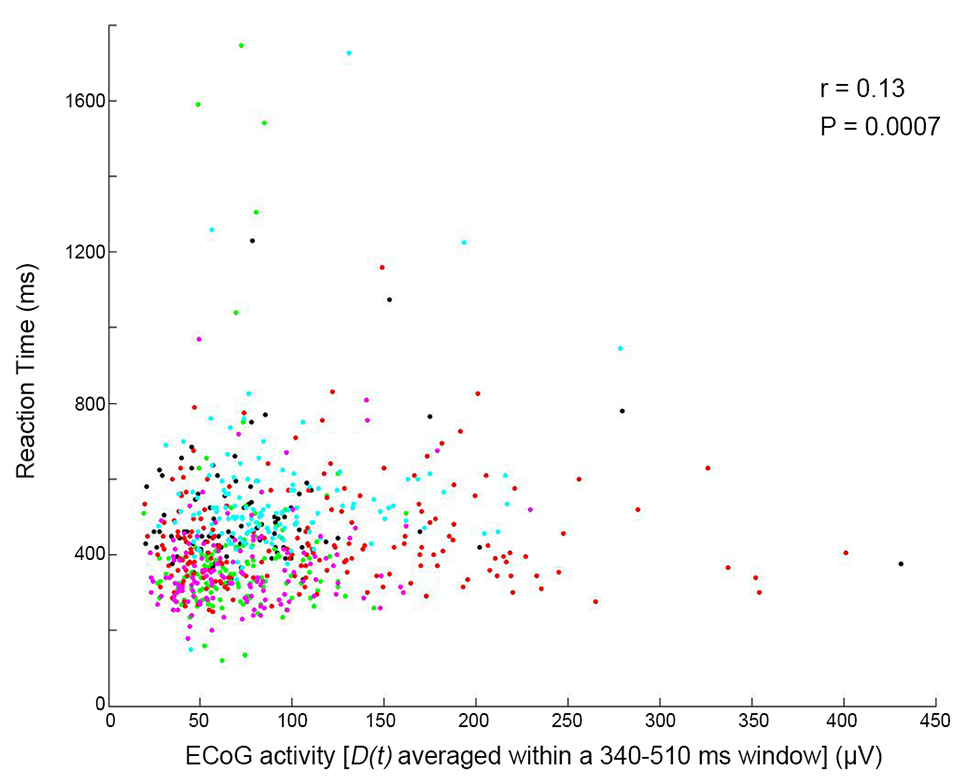

Supplement: Figure S2 — Correlation between RTs and D(t) across subjects. Related to Fig. 4B (top). The first PC was extracted from each subject's data (using contralateral hand). Its distance-to-mean D(t) time course was computed for each trial and averaged within a post-stimulus 340∼510 ms window around the behavioral response, then plotted against RT across all hit trials in all subjects. Different colors indicate different subjects. The Pearson correlation coefficient and associated P-value computed across all subjects are indicated in the graph. (TIF) [file pcbi.1003348.s002.tif]
