# Supplementary material for: Average Is Optimal: An Inverted-U Relationship between Trial-to-Trial Brain Activity and Behavioral Performance
Source: PLoS Comput Biol. 2013 Nov 7;9(11):e1003348. doi: 10.1371/journal.pcbi.1003348 (PMC3820514; doi:10.1371/journal.pcbi.1003348)
Supplement: Table S1 — Talairach coordinates (mm) of electrodes showing a significant linear or quadratic relationship with hit rate (left column) or RT (right column), as well as electrodes showing both relationships with hit rate or RT. Data from Patients #1, 2, 4 & 5 are included (see Fig. 6). Patient #3 is not included because the clinical CT scan was not obtained, thus the electrode locations in relation to MRI could not be determined. (DOCX) [file pcbi.1003348.s003.docx]

**Table S1.**

| **Hit Rate** | | | **RT** | | |
| --- | --- | --- | --- | --- | --- |
| *X* | *Y* | *Z* | *X* | *Y* | *Z* |
| **Linear** | | | **Linear** | | |
| -57.6 | -15.9 | 5.7 | -48.9 | 8.6 | 15.4 |
| -49.0 | -7.5 | 27.7 | -45.2 | 19.1 | 7.3 |
| -48.4 | 12.0 | 13.3 | -44.5 | -15.2 | 45.9 |
| -45.7 | 18.2 | 23.2 | 25.0 | -77.3 | 38.8 |
| 34.6 | -46.9 | 61.7 | 46.2 | -8.7 | 47.5 |
| 61.1 | -5.5 | 6.4 | 51.0 | -28.1 | 50.0 |
| **Quadratic** | | | 56.5 | -37.5 | 40.4 |
| -61.8 | -30.1 | 1.5 | 59.4 | -16.7 | 29.2 |
| -60.1 | -22.2 | -5.0 | 59.7 | -47.1 | 29.8 |
| -54.8 | -18.2 | 21.5 | **Quadratic** | | |
| -53.8 | -26.4 | 27.5 | -60.1 | -22.2 | -5.0 |
| -53.8 | -37.9 | 11.2 | -57.6 | -14.7 | -11.1 |
| -50.7 | -12.1 | 31.6 | -55.3 | -8.2 | -0.7 |
| -49.3 | -20.6 | 37.2 | -52.1 | -46.2 | 16.8 |
| -48.9 | 8.6 | 15.4 | -51.5 | -33.2 | 20.5 |
| -48.6 | -0.7 | -23.4 | -49.0 | -7.5 | 27.7 |
| -47.1 | 5.9 | -13.0 | -46.9 | 2.0 | 35.7 |
| -46.3 | -23.6 | 39.0 | -41.5 | -0.7 | 50.4 |
| -44.0 | 25.8 | 17.3 | 38.2 | -58.5 | 42.1 |
| 46.2 | -8.7 | 47.5 | 58.9 | -54.9 | -3.9 |
| 49.8 | -43.0 | 33.9 | 60.2 | -55.8 | 7.3 |
| 58.4 | -6.6 | 28.6 | **Both** | | |
| 60.2 | -55.8 | 7.3 | -49.8 | -41.4 | 26.1 |
| 63.4 | -34.9 | -4.2 | -41.2 | 24.0 | 33.3 |
| **Both** | | | -40.2 | 32.0 | 26.8 |
| -52.1 | -46.2 | 16.8 | 50.0 | -47.8 | 50.0 |
| -49.0 | 0.5 | 21.6 | 53.1 | -7.4 | 38.5 |
| 61.9 | -16.3 | 18.1 |  |  |  |
